# Digital social prescribing: a concept analysis

**DOI:** 10.3389/fpubh.2026.1857845

**Published:** 2026-07-01

**Authors:** Yiming Zhang, Anwei Xie, Ronghua Wang, Yulin Fan

**Affiliations:** 1 Department of Infectious Diseases, Children’s Hospital of Soochow University, Suzhou, China; 2 School of Nursing, Soochow University, Suzhou, China

**Keywords:** community health, concept analysis, digital health, digital social prescribing, integrated care, public health

## Abstract

**Introduction:**

Digital Social Prescribing (DSP) represents an innovative paradigm that integrates digital technologies into traditional social prescribing frameworks to address the social determinants of health (SDOH). However, persistent conceptual inconsistencies and a paucity of theoretical clarity have constrained its systematic application and evaluation within public health and nursing practices.

**Aims:**

This study aims to clarify the definition of digital social prescribing, distinguish it from traditional social prescribing, and identify its core characteristics through a systematic concept analysis.

**Methods:**

The Walker and Avant concept analysis framework was adopted. A comprehensive literature searches was conducted across multiple databases, including PubMed, CINAHL (EBSCOhost), APA PsycArticles, Scopus, Web of Science, Embase, IEEE Xplore, ACM Digital Library. A total of 30 relevant articles were included in the analysis. The analysis followed eight steps proposed by Walker and Avant: selecting concept, determining purposes of the analysis, identifying all uses of concept, determining defining attributes, constructing model and related cases, identifying antecedents and consequences, and defining empirical referents.

**Results:**

Digital social prescribing (DSP) is not merely a technological tool but a multidimensional public health model. Its five defining attributes are the uses of technology, non-clinical services, make personal plans based on needs, likes and location, community-based resources, and organizations from different sectors. The antecedents of DSP include contextual and population health drivers, medical systems and structural limitations, technological enabling factors, practice and implementation gaps and catalytic events and social development trends. The consequences of DSP encompass health and well-being outcomes, care and service delivery, system and implementation outcomes, equity and ethical considerations. Despite its potential benefits, DSP also faces challenges, including digital exclusion, data governance issues, gender imbalances and structural ethnic disparities.

**Conclusion:**

This study provides a comprehensive conceptual framework for digital social prescribing (DSP), addressing existing conceptual ambiguities and clarifying its theoretical boundaries. The study also highlights the need to critically address potential challenges associated with DSP implementation, including digital exclusion, data governance concerns, gender imbalances and structural ethnic disparities. These findings support the development of standardized evaluation frameworks and provide guidance for future research, practice and policy aimed at facilitating the sustainable and equitable implementation of DSP.

## 1 Introduction

In recent years, healthcare systems worldwide have increasingly adopted integrated care models that emphasize the interplay among biological, psychological and social determinants of health (1). Against this backdrop, social prescribing (SP) has emerged as an innovative intervention model designed to address individuals’ non-medical needs (2, 3). SP involves healthcare providers referring individuals with non-clinical needs to community resources and social services to address challenges such as social isolation, financial hardship, and insufficient support (4–6). Evidence suggests that social prescribing can improve mental well-being, promote social participation, and reduce pressure on healthcare services (2, 7).

Originating in the United Kingdom, social prescribing has evolved into a formalized service model characterized by multi-stakeholder collaboration (8, 9). This model is primarily grounded in social support theory and health behavior change theory (10). However, traditional social prescribing is constrained by several operational challenges, including reliance on manual referral workflows, fragmented information systems, and the lack of robust mechanisms for process monitoring and outcome evaluation. These limitations hinder the widespread adoption and effective implementation of SP (11, 12), often leading to inefficient resource utilization and missed opportunities for timely service provision. Previous studies have shown that referral inefficiencies may prevent eligible individuals from accessing social prescribing services, thereby limiting the potential system-level benefits of SP programs (13). Furthermore, population aging, the increasing burden of chronic diseases, and widening health inequalities underscore the urgent need for more efficient and coordinated healthcare delivery systems (14, 15). Against this backdrop, advances in digital technologies, including mobile health applications, electronic health records, and online service platforms, have facilitated the emergence of digital social prescribing (DSP) as an innovative service model (16). By integrating digital technologies into core processes such as needs assessment, resource matching, service referral and outcome monitoring, DSP enhances service efficiency, facilitates personalized care delivery, and expands access to community-based health resources (17, 18). DSP represents not only a technological extension of conventional social prescribing but also a fundamental transformation of its operational framework (19).

Despite the growing adoption of digital social prescribing (DSP), no consensus has yet been reached regarding its definition. Some studies characterize DSP primarily as a digital solution for streamlining referral workflows (20), whereas others conceptualize it as a holistic integrated care model that combines healthcare services with social support interventions (21). Furthermore, the implementation of DSP varies considerably across healthcare settings. For example, a large-scale study conducted within the OCHIN network found that many clinics had not activated the social-needs coordination functionalities available within their electronic health record systems, indicating substantial variation in how DSP is interpreted, operationalized, and integrated into routine clinical practice across organizations. Such variation further underscores the lack of conceptual clarity surrounding DSP. In addition, the inherent tension between algorithm-driven automated matching systems and person-centered service models continues to blur the conceptual boundaries (22). These inconsistencies limit the comparability of research findings and hinder the development of standardized evaluation frameworks.

Although DSP is generally regarded as an extension of traditional social prescribing, it remains unclear whether, and to what extent, its digitalized processes align with the core principles of social prescribing. According to the international consensus articulated by Muhl et al. (23), a valid social prescribing model should satisfy the Common Understanding of Social Prescribing (CUSP) framework, which comprises four sequential conditions: recognizing non-clinical needs, providing a linking pathway, co-producing a personalized plan, and generating multidimensional health and well-being benefits. However, although these conditions establish the conceptual foundation of traditional social prescribing, the ways in which they are reconstructed, augmented, and operationalized within digital ecosystems remain insufficiently theorized. Therefore, drawing on Walker and Avant’s concept analysis methodology (24), this study employs the CUSP framework as a guiding theoretical lens to systematically examine digital social prescribing (DSP). Specifically, this study addresses two key research questions: (1) What is the conceptual meaning of DSP as an evolution of traditional social prescribing? and (2) What are its defining attributes, antecedents, and consequences within contemporary public health and nursing practice? By clarifying how digitalization reshapes the underlying logic of social prescribing, this study seeks to provide a robust theoretical foundation for future empirical research in digital health and for the advancement of evidence-based nursing practice.

## 2 Methods

### 2.1 Concept analysis method

Concept analysis is a way to deconstruct a systematic method for deconstructing a concept to enhance understanding and develop a rigorous definition that facilitates its measurement and empirical application (25). Given that digital social prescribing (DSP) relies heavily on digital information systems and standardized clinical pathways, its conceptualization requires a high degree of conceptual precision, operational clarity, and definitional rigor. Walker and Avant’s concept analysis framework (24) is particularly well suited to this study because of its highly structured eight-step approach. By systematically identifying antecedents, consequences, and empirical referents, this framework provides a robust methodological foundation for the conceptual clarification and standardized implementation of DSP across clinical practice and digital healthcare systems.

The steps are as follows: (1) Select a concept; (2) Determine the aims or purposes of analysis; (3) Identify all uses of the concept that you can discover; (4) Determine the defining attributes; (5) Identify a model case; (6) Identify borderline, related, contrary, invented cases; (7) Identify antecedents and consequences; (8) Define empirical referents (24).

### 2.2 Data sources

A systematic literature search was conducted across 8 electronic databases, including PubMed, CINAHL (EBSCOhost), APA PsycArticles, Scopus, Web of Science, Embase, IEEE Xplore, ACM Digital Library from database inception to May 2026. To ensure a comprehensive retrieval of the conceptual boundaries of Digital Social Prescribing (DSP), we employed a combination of Boolean operators and the following keywords: (“Digital” OR “eHealth” OR “Online” OR “Web-based” OR “electronic health” OR “digital medicine” OR “digital health”) AND (“Social Prescribing” OR “social prescription*” OR “social prescribe*”). Guided by Walker and Avant’s conceptual framework, literature published in English was broadly retrieved across multiple disciplines and formats to accurately map the concept’s attributes. Detailed search strategy can be found in Supplementary Table 1.

### 2.3 Data collection

The inclusion criteria were: (1) Regarding the core attributes, antecedents and consequences of DSP; (2) Has been officially published; (3) Language is English. The exclusion criteria included: (1) Literature that focuses solely on digital health but lacks the connotation of connecting community resources; (2) Literature that cannot be accessed in full or has duplicate publication of data. The study selection process was conducted in accordance with the PRISMA 2020 guidelines (26). Initially, all retrieved records were imported into EndNote 21 for duplicate removal. Two reviewers independently screened the titles and abstracts according to the predefined eligibility criteria. Subsequently, the full texts of the potentially eligible articles were retrieved and independently assessed for inclusion. Any disagreements between the reviewers were resolved through discussion, and when necessary, consultation with a third senior researcher. Ultimately, 30 articles met the inclusion criteria and were included in the concept analysis. The screening process is shown in Supplementary Figure 1. Summary of articles is shown in Supplementary Table 2.

### 2.4 Data analysis

Data analysis was conducted in accordance with Walker and Avant’s eight-step concept analysis framework (24). To enhance analytical rigor, two researchers independently reviewed the included articles multiple times to develop a comprehensive understanding of DSP. A standardized data extraction matrix was developed to systematically capture definitions, characteristics, contextual factors, outcomes, and measurement indicators reported in the literature. The extracted data were compared across studies to identify recurring patterns, conceptual similarities, and distinguishing features. Characteristics that were consistently reported across studies and distinguished DSP from related concepts were synthesized into defining attributes. Based on these defining attributes, model, borderline, related, and contrary cases were systematically constructed to clarify the conceptual boundaries of DSP. Factors identified as prerequisites for the occurrence or implementation of DSP were categorized as antecedents, whereas outcomes resulting from DSP were classified as consequences. Empirical referents were subsequently identified to determine how the concept could be operationalized, observed, and measured in practice. Any discrepancies arising during the iterative analytical process were resolved through discussion until consensus was achieved. Finally, an operational definition of DSP was developed through the synthesis of its defining attributes, while antecedents, consequences, and empirical referents were used to further refine and contextualize the concept.

## 3 Results

### 3.1 Uses of the concept

Digital Social Prescribing has been widely discussed in the literature, with studies emphasizing different functions and scope of application (20, 21). Some studies conceptualize DSP as a digital referral tool that utilizes online platforms or electronic systems to identify social needs and connect individuals with appropriate community services (20). Within this perspective, DSP primarily aims to enhance referral efficiency and facilitate communication between healthcare providers and community organizations (27, 28).

In contrast, other studies define DSP as a comprehensive care model in which digital technologies are embedded within an integrated system that connects healthcare services with social and community resources (21, 22). Within this conceptualization, DSP extends beyond a mere technological tool (29) and functions as a structured service model that relies on cross-sector collaboration and continuous care coordination to address the social determinants of health (30, 31).

This conceptual diversity contributes to ambiguity regarding the defining attributes and conceptual boundaries of DSP, thereby underscoring the need for systematic concept clarification.

### 3.2 Dictionary definition

To establish a foundation for concept analysis, the term digital social prescribing (DSP) was first examined from a linguistic perspective. A review of major lexicographical sources, including the Oxford English Dictionary, Cambridge Dictionary, and Collins Dictionary, indicates that the term digital refers to the use of computer technologies, electronic systems, and internet-based platforms for information processing, whereas social prescribing refers to the practice of referring individuals to non-clinical community services to improve health and well-being. From an etymological perspective, DSP can be broadly understood as the application of digital technologies to facilitate the identification of non-clinical needs, the connection of individuals to community resources, and the coordination of support services. However, dictionary-based definitions provide only a preliminary linguistic interpretation and fail to capture the socio-technical complexity of DSP within contemporary integrated care systems.

### 3.3 Defining attributes

Guided by the international consensus framework (CUSP) (23) and the realist implementation pathway conceptualized by Husk et al. (32), this study redefines the five core attributes of DSP “use of technology; non-clinical services; Make personal plans based on needs, likes and location; Community-based resources; Organizations from different sectors” by embedding them into three distinct chronological stages: Enrolment, Engagement, and Adherence. These attributes not only distinguish DSP from traditional biomedical interventions but also differentiate it from non-digital social prescribing approaches.

#### 3.3.1 Use of technology

Digital platforms, mobile applications, and electronic referral systems (21, 33) are widely employed within social prescribing pathways to support resources identification, referral management, process monitoring, and outcome evaluation (17, 34–36). These technologies not only enhance information exchange between healthcare providers and community organizations but also facilitate continuous monitoring of the referral process and service engagement (19, 30, 31, 37–41). Rather than functioning merely as a discrete tool, technology in DSP serves as a dynamic infrastructure that reconfigures the entire service continuum (42), aligning with the realist pathway of enrolment, engagement, and adherence proposed by Husk et al. (32). During the enrolment phase, this attribute is manifested through the algorithmic identification of non-clinical needs (28, 43). However, empirical evidence highlights an inherent tension between standardized digital documentation and narrative clinical workflows, where time-constrained link workers heavily favor unstructured narrative notes over rigid, standardized digital drop-down menus (13, 42). During the engagement phase, this attribute is characterized by system-native integration, such as embedded electronic health record (EHR) modules, combined with patient-facing automation (27, 44). Nevertheless, this phase reveals a paradox in which high system availability does not necessarily translate into widespread clinical adoption, illustrating that the technical presence of embedded modules does not automatically guarantee active clinical utilization (44). During the adherence phase, technology functions as a closed-loop feedback mechanism that enhances the visibility of service engagement and outcomes (27, 45). Nonetheless, the effectiveness of this technological attribute is constrained by implementation limitations; Evidence suggests that digital dashboards and automated communication channels alone are insufficient to motivate human action or sustain long-term adherence in the absence of coordinated interpersonal support (44, 46).

#### 3.3.2 Non-clinical services

Unlike conventional interventions that primarily rely on medical treatment, DSP connects individuals with a wide range of community-based activities and social support services (13, 17, 21, 37, 47). These services include sports programs, arts and cultural activities, peer support groups and welfare counseling services (19, 33, 48), which are intended to address loneliness, social isolation, and other social and psychological factors that adversely affect health and well-being (16, 34, 35, 41, 49, 50).

Within the DSP framework, this attribute extends beyond a static catalog of non-clinical options and is manifested through the digital codification, aggregation, and dynamic mapping of fragmented local resources (28, 43). From the realist perspective proposed by Husk et al. (32), this attribute serves as a key mechanism for promoting sustained engagement and long-term adherence (27). In traditional social prescribing, access to non-clinical services is often constrained by fragmented and asymmetrical information regarding available community resources. In DSP, however, this attribute is redefined through enhanced resource visibility and the ability to monitor service utilization and outcomes (45). Consequently, community-based social activities are transformed into structured, digitally navigable interventions that can be integrated into broader health promotion strategies (43, 45). Within this framework, sustained engagement is driven not by clinical compliance alone but by the alignment between individuals’ unmet social needs and the perceived well-being benefits derived from community-based interventions (29, 42).

#### 3.3.3 Make personal plans based on needs, likes, and location

During implementation, healthcare professionals usually assess an individual’s health status (28, 29, 47), social needs, interests, preferences (41, 42), and living environment to develop personalized social prescription plans (35). Digital technologies facilitate this process through resource-matching systems that recommend tailored community services based on individuals’ needs and geographic locations (28, 33, 43). This person-centered approach enhances the precision of service matching and supports sustained engagement in community-based activities (28, 29).

Beyond reflecting the co-production principles of traditional social prescribing, this attribute functions as a key mechanism during the enrolment stage of the realist pathway proposed by Husk et al. (32). According to the realist perspective, initial reluctance to engage often arises from perceived mismatches between available community resources and personal circumstances, as well as uncertainty about navigating unfamiliar non-clinical referral pathways (45). Within DSP, this attribute is characterized by data-driven personalization and contextual responsiveness, transforming traditional manual signposting into an automated and highly individualized matching system (28, 43, 45). By systematically aligning local community resources with an individual’s socioeconomic circumstances, personal interests, and geographic context, this attribute reduces uncertainty and strengthens trust in the referral process (28, 45). By replacing administrative ambiguity with contextually relevant recommendations, it creates a low-barrier and accessible entry point that facilitates initial enrolment and promotes a seamless transition into the broader service continuum (27, 28, 45).

#### 3.3.4 Community-based resources

DSP relies on existing community resources (28, 37, 39), including community organizations, volunteer institutions, and a wide range of social service agencies (27, 33). By connecting individuals with these community resources (34–36, 43), DSP extends social support beyond the conventional healthcare system and encourages active participation in community life (16, 29). This community-based intervention approach contributes to the development of a more comprehensive and sustainable network of health and social support (27, 33, 35, 47). Within DSP, this attribute extends beyond traditional signposting and is operationalized through the digital coordination and deployment of community resources to address multiple social determinants of health (SDOH) (28, 43). DSP platforms reconfigure community resources by transforming previously fragmented and isolated services into interconnected, digitally searchable networks that include food assistance, financial counseling, housing support, and peer support groups (27, 28, 42). Importantly, this attribute is characterized by a dual dynamic: it enhances cross-sector service coordination while simultaneously exposing challenges related to equitable access and engagement (46). Empirical evidence suggests that although digital infrastructures can effectively mobilize community resources for many users, they remain less effective for digitally excluded or clinically complex populations, indicating that digital resource integration alone cannot overcome entrenched socioeconomic and access-related barriers (29, 46).

#### 3.3.5 Organizations from different sectors

Through collaboration among healthcare institutions (34, 35), community organizations, volunteer groups, and social service agencies (27, 28, 36), healthcare and social resources can be effectively integrated (37, 43, 45). This integration provides more holistic support for individuals and contributes to the development of an integrated system that combines healthcare and social services (17, 29, 35, 41). The supplementary information about the attributes is shown in Supplementary Table 3.

When considered together, the attributes of community-based resources and cross-sector interorganizational collaboration form the structural foundation of multi-agency coordination in DSP, corresponding closely to the engagement stage of the realist pathway proposed by Husk et al. (32). Traditional social prescribing frequently experiences operational challenges and high rates of participant attrition during the “supported transition” phase, a critical handoff period between primary care services and community-based resources (27, 45). Within the DSP paradigm, these attributes help mitigate transitional barriers through interoperable systems and continuous information exchange across organizational boundaries (45). Rather than relying on manual, paper-based, or isolated referral processes, DSP operationalizes a networked infrastructure supported by secure electronic referral platforms and automated communication systems (27, 28, 42). This infrastructure establishes a traceable digital connection between healthcare professionals and community resource coordinators (43, 45). Consequently, cross-sector digital integration enables continuous referral tracking and coordination, transforming historically fragmented referral processes into a synchronized and secure multi-agency care ecosystem that reduces disengagement during transitions between sectors (27, 28, 45).

### 3.4 Antecedents

Antecedents refer to the pre-existing events and contextual conditions that lay the foundational background and driving forces for the formation of a specific concept. Identifying these antecedents is critical to understanding the contextual background and structural motivations underlying concept emergence (24). Through a comprehensive analysis of relevant literature, the antecedents of DSP can be summarized into five core aspects: contextual and population health drivers, medical systems and structural limitations, technological enabling factors, practice and implementation gaps, and catalytic events and social development trends.

#### 3.4.1 Contextual and population health drivers

Health outcomes are substantially influenced by social determinants of health (SDOH) (27, 28, 34), including loneliness, social isolation, housing instability, and food insecurity (27, 28, 45). These needs are often inadequately addressed within traditional healthcare systems (16, 29, 33, 40, 43, 45). Furthermore, persistent health inequalities, together with demographic shifts such as population aging and the growing burden of chronic diseases, have intensified the demand for integrated and community-oriented models of care (35, 37, 38, 47).

#### 3.4.2 Medical systems and structural limitations

Furthermore, older adults living with multimorbidity often face compounded financial and caregiving burdens that are inadequately addressed within traditional primary care systems (29, 35, 51). Contemporary healthcare systems continue to be largely shaped by biomedical models that prioritize diagnosis and clinical treatment while paying limited attention to the broader social determinants of health (13, 34, 43). Within these fragmented models of care, systematic screening for social determinants of health (SDOH) is often absent from routine clinical practice, limiting the timely identification of non-medical needs (27, 28, 30). This limitation is further compounded by time constraints in frontline clinical settings, where brief consultation periods reduce opportunities to identify social needs and contribute to the underutilization of social prescribing referrals among eligible individuals (13, 27).

#### 3.4.3 Technological enabling factors

Technological enabling factors have provided essential foundations for DSP (19, 29, 35, 41). Technologies such as mobile health applications, electronic health records (EHRs) and cloud-based platforms facilitate automated referrals, information exchange, and service coordination (21, 27, 28, 33, 35, 46). Traditionally, the primary functions of electronic health record systems have been limited to clinical documentation and administrative billing (43). However, their potential to streamline clinical workflows and provide patients with actionable information regarding community and social resources remains underutilized (43).

#### 3.4.4 Practice and implementation gaps

Beyond broader structural limitations, operational challenges within traditional non-digital social prescribing models have further highlighted the need for digital alternatives (27, 36, 37, 45). Conventional social prescribing programs often rely on manual, paper-based referral processes and are characterized by limited interoperability between healthcare institutions and community organizations (36, 37, 45). Such fragmentation constrains scalability, increases the risk of participant disengagement during transitions between organizations, and limits the capacity for real-time referral tracking and outcome monitoring (17, 42, 45).

#### 3.4.5 Catalytic events and social development trends

Social disruptions and emerging public health challenges have accelerated the development and adoption of DSP (19, 29, 35, 39). One of the most significant catalysts was the COVID-19 pandemic, which necessitated physical distancing measures and accelerated the transition of healthcare systems toward integrated and digitally enabled models of care (35, 46). This societal shift exposed and intensified existing public health vulnerabilities. For example, pandemic-related restrictions highlighted challenges related to adolescent sexual and reproductive health (SRH), while also exacerbating social isolation among individuals living with chronic pain, thereby increasing vulnerability to adverse mental health and substance-use outcomes (50). Collectively, these challenges underscored the limitations of conventional healthcare delivery models and highlighted the need for digitally enabled approaches capable of connecting healthcare services with community-based support systems (28, 43, 45). The supplementary information about the antecedents is shown in Supplementary Table 4.

### 3.5 Consequences

Consequences refer to the results produced by the implementation or development of a certain concept (24). In the context of Digital Social Prescribing (DSP), its consequences manifest as various impacts at the health, service, and social levels, which can be either positive or potentially risky.

#### 3.5.1 Health and well-being outcomes

Research demonstrates that DSP can improve physical and mental health (19, 29, 33, 35), enhance social connections, and strengthen individuals’ self-management capabilities (34, 35, 43, 47). Several empirical studies provide support for these reported benefits. For example, a large-scale real-world study involving children and adolescents reported a mean increase of 3.72 points in psychological well-being scores following participation in a DSP intervention, indicating a clinically meaningful improvement (42). Similarly, among adults with hypertension, participation in a DSP-supported intervention was associated with an increase in blood pressure control rates from 0% at baseline to 48.3% after one year of follow-up (27).

In addition to these clinical outcomes, DSP may also facilitate sustained engagement in health-promoting activities through continuous monitoring, feedback, and coordinated support services (27, 45). By strengthening connections between individuals and community-based resources, DSP may enhance perceived care value, encourage ongoing participation, and support long-term adherence to recommended interventions (29, 42).

Despite these benefits, the literature also highlights several challenges and unintended consequences associated with DSP, including digital fatigue, reduced opportunities for face-to-face interaction, and lower levels of long-term engagement among some service users (16, 37, 45). These findings suggest that the effectiveness of DSP may vary across populations and settings and is influenced by the extent to which digital approaches are successfully integrated with human support and community-based care (29, 46).

#### 3.5.2 Access to care and service delivery

DSP improves access to community resources, facilitates personalized referrals, and enhances coordination between health and social care services (28, 35, 36). By integrating digital platforms into referral and care coordination processes, DSP can streamline service delivery and reduce barriers to the identification and management of non-clinical needs (27, 28).

Empirical evidence supports these service-level improvements. For example, one established DSP program screened 11,819 pediatric patients over a 14-month period, achieving a screening rate of 73.5% among the target population (44). In addition, DSP-enabled coordination mechanisms have shown promise in addressing urgent social determinants of health (SDOH) needs, with studies reporting that up to 87% of individuals successfully had their acute social needs addressed through connections with appropriate community resources (27, 29).

Despite these improvements, the literature suggests that these benefits may not be distributed equitably across all population groups. Vulnerable populations, including older adults, individuals with multimorbidity, and those with limited digital literacy, may encounter difficulties engaging with digitally mediated services (38, 39, 46). Consequently, although DSP has the potential to expand access to community-based support and improve service delivery efficiency, it may inadvertently exacerbate disparities in service utilization if issues related to digital accessibility, usability, and inclusiveness are not adequately addressed (29, 46).

#### 3.5.3 System and implementation outcomes

At the system level, DSP may contribute to more efficient resource allocation, improved service delivery, and enhanced collaboration among healthcare providers, community organizations, and social service agencies (28, 34, 35, 40). Through the integration of digital platforms into referral and coordination processes, DSP facilitates information sharing and enhances organizational capacity to respond to individuals’ non-clinical needs in a timely and coordinated manner (27, 28).

Beyond organizational efficiency, DSP may also generate broader community-level benefits. For example, one study reported that approximately 48% of individuals who received automated community resource information subsequently shared these resources with family members, friends, or other social contacts (43). Such diffusion extends the reach of community support services beyond direct recipients and may increase community awareness of available health and social resources (43).

Overall, DSP has the potential to strengthen system integration, enhance interorganizational collaboration, and promote more efficient utilization of community resources, thereby improving the responsiveness and sustainability of health and social care systems (13, 29, 45).

#### 3.5.4 Equity and ethical considerations

Although DSP has the potential to reduce health inequalities and strengthen community service capacity, several challenges remain, including digital exclusion, the medicalization of social services, and variations in implementation quality across regions (13, 17, 19, 34, 35). One major concern is the persistence of digital exclusion, whereby individuals with limited digital literacy, inadequate internet access, socioeconomic disadvantage, or older age may encounter barriers to engaging with digitally enabled services (29, 46). Consequently, the benefits of DSP may not be equitably distributed across all population groups.

Furthermore, the growing reliance on digital platforms and algorithm-assisted referral processes raises concerns regarding fairness, transparency, accountability, and potential bias in service delivery (42, 45). Differences in local infrastructure, resource availability, and implementation capacity may further contribute to geographic disparities in access to DSP services (44). Additionally, some scholars have argued that integrating social needs into healthcare systems may inadvertently contribute to the medicalization of social problems, potentially blurring the distinction between healthcare and social care (13, 43). Collectively, these findings indicate that although DSP offers substantial opportunities to improve health and social outcomes, careful attention to equity, inclusion, and ethical governance is necessary to ensure that digitally enabled services do not inadvertently reinforce existing social and health disparities (29, 35, 46). The supplementary information about the consequences is shown in Supplementary Table 5. Antecedents, attributes and consequences are shown in Supplementary Figure 2.

### 3.6 Cases

Identifying cases enables researchers to apply the defining attributes of a concept to practical scenarios and distinguish whether relevant cases are identical, associated, similar or distinct to the original concept (24). Examining additional cases further facilitates in-depth discussion and helps clarify which attributes best represent the concept. Model, borderline, related, contrary, and invented cases from the Walker and Avant framework are used to interpret the defining attributes of DSP.

#### 3.6.1 Model case

A model case includes all the defining attributes of a concept, thereby clearly demonstrating its existence (24).

Mr. Smith suffers from social isolation and frequent hospital visits. Recognizing his non-clinical needs, his GP uses a DSP platform to accurately match him with a nearby, volunteer-run community gardening group. The link worker completes his digital registration on a tablet, while the platform syncs with the group’s check-in system to monitor participation and tracking outcomes in real time. After two months, his social participation increased and unnecessary clinic visits dropped. This case illustrates all the defining attributes of Digital Social Prescribing.

#### 3.6.2 Borderline case

Borderline cases include most of the defining attributes of a concept but lack one or more key characteristics, helping to clarify how the absence of specific attributes alters the meaning of the concept (24).

Grandma Jin suffers from social isolation and cognitive impairment. Her GP utilizes a DSP platform to input her health and location data, and the system automatically matches her with a local welfare center’s weekly craft workshop. The platform sends navigation routes and electronic vouchers to her smartphone. However, Grandma Jin’s low digital literacy prevents her from using the app, causing her to miss the activity. Although technology identified her non-clinical needs, the actual connection to community-based resources failed.

#### 3.6.3 Related case

Related cases describe concepts that are connected to the concept being studied but lack its defining attributes. They help clarify how the concept relates to other similar or overlapping concepts (24).

During a hypertension follow-up, Mr. Li’s GP focuses strictly on biomedical management, ignoring his social isolation. The GP instructs him to use a hospital mHealth app that automatically reminds him to reduce salt and tracks home exercises. Mr. Li uses this digital tool independently to manage his clinical condition. The hospital mHealth app offers no links to community resources. It has no cooperation with organizations from different sectors. It also fails to assess non-clinical services, make personal plans based on needs, likes and location.

#### 3.6.4 Contrary case

Contrary cases are examples that contain none of the defining attributes of the concept. They help clarify the boundaries of the concept and distinguish it from unrelated cases (24).

During a routine clinic visit for chronic conditions, Mr. Taylor’s GP adjusts his medication, prints a paper prescription, and provides brief verbal lifestyle advice. Throughout the process, the clinician uses no digital health tools, ignores the patient’s social determinants, and has no cooperation with local community or charitable organizations. Mr. Taylor leaves the clinic alone with his prescription. This case does not contain any definitional attributes of the digital healthcare prescription. It falls under the category of typical traditional medical practice.

### 3.7 Empirical referents

Empirical referents are observable indicators that demonstrate the presence of a concept in practice. They provide measurable evidence for the defining attributes and support the operationalization of the concept in research and healthcare settings (24). For Digital Social Prescribing (DSP), empirical referents reflect the practical and measurable manifestations of its defining attributes within digitally enabled and community-oriented healthcare systems.

The attribute of “Uses of technology” is evidenced by observable digital behaviors, such as the frequency of logins (46), duration of platform engagement (42), and the responsiveness of users to automated notifications (46, 52). While the eHealth Literacy Scale (eHEALS) provides a measure of perceived digital competence (29, 53).

The provision of non-clinical services manifests as observable shifts in social participation (42). These referents include the number of new community connections made and the rate of attendance at community-led activities (29). These can be quantified using the Social Prescribing Wellbeing Outcomes Framework (SWOF), which measures dimensions of social connectivity and psychosocial well-being (42, 54).

The “making personal plans based on needs, likes, and location” attribute is recognized when care plans explicitly reflect a patient’s unique social needs, preferences, and geographic location (28). This is empirically assessed through qualitative audits of care records or the Individualized Care Scale (ICS) (42, 46), which evaluates whether the care provided matches the patient’s personal context (55).

The presence and accessibility of community-based resources may be identified through community asset mapping, which documents local organizations, services, and support networks within a defined geographical area (27, 28, 43). Finally, organizations from different sectors, an essential component of DSP, may be evaluated using the Partnership Self-Assessment Tool (PSAT) (35, 45, 56), which assesses partnership functioning, coordination, and resource sharing among collaborating organizations (27, 35). The supplementary information about the Empirical referents is shown in Supplementary Table 6.

## 4 Definition

Following the analysis of its attributes, antecedents, and consequences, DSP refers to a technology-enabled approach that facilitates personalized referrals to non-clinical community resources according to their needs, preferences, and geographical contexts. Through digital platforms and cross-sector collaboration between healthcare providers and community organizations, DSP addresses social determinants of health and improves population health and overall well-being.

## 5 Discussion

This study conceptualizes Digital Social Prescribing (DSP) as a multi-dimensional socio-technical public health model rather than a simple digital referral tool. The core contribution lies in demonstrating that DSP integrates digital infrastructure, social determinants of health, and community-based resources into a coordinated system that reshapes the traditional logic of social prescribing. While DSP enhances accessibility, efficiency, and system responsiveness, it simultaneously exposes persistent structural inequalities, indicating that technological advancement alone is insufficient to achieve health equity.

### 5.1 Principal findings and theoretical contribution

The five core attributes in this study: Uses of technology; Non-clinical services; Make personal plans based on needs, likes and location; Community-based resources; Organizations from different sectors. Clarifying these attributes advances existing understandings of social prescribing by distinguishing DSP from related concepts such as telehealth services, electronic referral systems, and broader digital health interventions (35, 45). Unlike these technology-centered approaches, DSP explicitly focuses on addressing multidimensional social determinants of health (SDOH) through coordinated engagement with community-based resources and non-clinical support services (13, 16, 17).

Importantly, these findings extend the Common Understanding of Social Prescribing (CUSP) framework proposed by Muhl et al. (3) by demonstrating how its foundational principles are operationalized within digital environments (42). In DSP, the identification of non-clinical needs is increasingly supported through digital screening and assessment mechanisms (27, 28, 46). Linking pathways are transformed into automated and traceable referral processes facilitated by digital platforms (27, 28, 45), while personalized plans are strengthened through data-informed tailoring and continuous monitoring (27, 28, 42). In addition, multidimensional health and well-being outcomes are supported through coordinated deployment of community-based assets and services (29, 43). Therefore, DSP should be understood as a technological evolution of social prescribing rather than a replacement of its foundational principles.

Furthermore, by integrating the realist pathway proposed by Husk et al. (32), this study provides a socio-technical framework for understanding how digital technologies support engagement across different stages of the social prescribing pathway. During the enrolment stage, personalized planning and location-based matching may function as trust-building mechanisms by reducing uncertainty and improving the relevance of recommended services to individual circumstances (28, 43). During the engagement stage, the integration of community-based resources and cross-sector interorganizational collaboration facilitates informational continuity and coordinated service delivery, thereby reducing the risk of disengagement during transitions between healthcare and community settings (27, 43). During the adherence stage, digital technologies support ongoing monitoring and feedback through mechanisms such as digital dashboards and referral tracking systems, helping to reinforce the perceived value of non-clinical interventions and sustain long-term participation (45).

### 5.2 Empirical support for DSP effectiveness

The conceptual framework developed in this study provides a theoretical foundation for understanding the positive outcomes associated with DSP reported across previous empirical studies. Evidence from diverse healthcare settings suggests that DSP may improve mental well-being (29), support chronic disease management (43), enhance social connectedness (29), and strengthen self-management capacity (35). These benefits may be attributable, in part, to DSP’s capacity to integrate community resources with digital monitoring and feedback mechanisms, thereby supporting sustained engagement with non-clinical interventions.

At the service level, DSP appears to improve the identification of social needs (28), facilitate timely referrals (27), and strengthen coordination among healthcare providers, community organizations, and social service agencies (35). The integration of digital platforms into referral pathways may reduce information fragmentation, improve communication across sectors (28), and enhance the operational efficiency of social prescribing programs. Collectively, these findings suggest that DSP represents not only a conceptual advancement but also a promising practice model for addressing social determinants of health within contemporary healthcare systems.

Nevertheless, the effectiveness of DSP is likely to be influenced by implementation quality, the availability of community resources, and the extent to which digital systems are successfully integrated into routine healthcare practice (28). Consequently, the benefits and limitations of DSP should be considered within their broader organizational, technological, and social contexts.

### 5.3 Equity challenges and structural limitations

Despite its potential benefits, DSP does not inherently reduce health inequalities. The findings suggest that digital technologies may simultaneously enhance access for certain populations while creating new barriers for others. Factors such as limited digital literacy, inadequate internet access, socioeconomic disadvantage, and age-related barriers may hinder engagement with digitally enabled services, thereby limiting the equitable distribution of DSP benefits.

In addition, evidence indicates that substantial implementation challenges remain even when digital referral infrastructures are available. Variations in adoption, utilization, and engagement across healthcare settings suggest that technological availability alone does not guarantee effective integration into routine clinical workflows (27, 28, 44, 45). These findings underscore the importance of organizational readiness, workforce capacity, and implementation support in shaping the successful integration of DSP into routine practice.

Furthermore, disparities in participation and service utilization across demographic and social groups raise concerns that existing social and health inequities may be replicated within digitally mediated referral pathways (49, 52). The increasing reliance on digital platforms and algorithm-assisted decision-making processes also raises important questions regarding transparency, accountability, and fairness in service delivery (29, 42). If left unaddressed, these challenges may result in digital systems inadvertently reinforcing, rather than alleviating, existing inequalities.

Collectively, these findings indicate that DSP should not be conceptualized as a purely technological solution (29, 46). Rather, its effectiveness and equity depend on how digital infrastructures interact with broader social, organizational, and community contexts (44). Accordingly, maintaining human-centered care, professional judgment, and relational continuity remains essential to ensuring that digital innovation supports, rather than replaces, the foundational values of social prescribing.

### 5.4 Implications for practice and policy

The findings suggest that DSP should be implemented as a socio-technical system rather than a technology-driven intervention, as its effectiveness and equity depend on the interaction between digital infrastructures, healthcare organizations, community resources, and service users (28, 29, 46). Technological innovation alone is unlikely to achieve the intended benefits of DSP without supportive organizational structures, sustainable community partnerships, and equitable implementation strategies (43, 46).

First, interoperable digital infrastructures and robust data governance frameworks are essential to facilitate information sharing, improve service coordination, and support continuity of care across healthcare and community sectors (27, 45, 46). Digital systems should be designed to integrate seamlessly into existing clinical workflows while minimizing administrative burden for healthcare professionals. Second, the findings highlight the continued importance of human support within digitally enabled service models (29, 46). Although digital platforms can improve efficiency and scalability, successful engagement with community resources often depends on personalized guidance and ongoing support (46). Therefore, digital technologies should complement rather than replace the role of link workers and other frontline professionals who facilitate navigation across health and social care systems. Third, equity considerations should be embedded throughout the design and implementation of DSP programs. Targeted strategies to improve digital literacy, enhance accessibility, and reduce technological barriers are particularly important for older adults, rural populations, and socioeconomically disadvantaged groups. Without such efforts, digital innovations may inadvertently reinforce existing health and social inequalities.

Importantly, DSP should remain grounded in a human-centered care model that preserves professional judgment, relational continuity, and individualized support alongside digital efficiency (30, 51). Future research should further examine the contextual factors influencing DSP implementation across different healthcare systems and population groups, with particular attention to long-term equity outcomes, mechanisms of sustained engagement, and the interaction between digital infrastructures and human support systems. Such evidence will be critical for informing the sustainable and equitable development of DSP in future health and social care practice.

## 6 Limitations

This concept analysis has several limitations. First, the included literature was restricted to English-language publications, which may have resulted in the exclusion of relevant evidence reported in other languages. Second, as DSP is an emerging and rapidly evolving concept, the included studies exhibited substantial heterogeneity in terminology, research design, healthcare contexts, and technological platforms, which may have influenced the interpretation and synthesis of conceptual elements. Third, although rigorous analytical procedures were employed, concept analysis inherently involves a degree of interpretive judgment in the identification and synthesis of defining attributes, antecedents, consequences, and empirical referents. Therefore, some level of subjectivity cannot be entirely eliminated. Finally, the findings are primarily theoretical in nature. As DSP continues to evolve alongside developments in digital health technologies and healthcare systems, the conceptual framework proposed in this study should be further validated and refined through future empirical research across diverse settings and populations.

## 7 Conclusion

In conclusion, guided by the Walker and Avant concept analysis framework, this study systematically clarifies the concept of Digital Social Prescribing (DSP). The core attributes identified encompass uses of technology; the provision of non-clinical services; Make personal plans based on needs, likes and location; Community-based resources; Organizations from different sectors. By refining the conceptual boundaries and theoretical implications of DSP, this study elucidates how digital tools empower social prescribing practices and mitigate adverse social determinants of health. This conceptual clarification provides a foundation for future empirical investigations and supports the formulation of evidence-based interventions and policy initiatives to facilitate the systematic integration Digital Social Prescribing into healthcare systems.

## 8 Implications for research

Research Implications: As an emerging concept, DSP still requires empirical validation in various medical systems. Future research should prioritize evaluating its effectiveness in improving health outcomes and social connections and develop standardized measurement tools to assess its sustainability and fairness. Additionally, it is crucial to clarify the impact of different digital platforms and referral mechanisms on intervention effects through comparative studies.

Practical and Policy Insights: At the practical level, clear conceptualization is conducive to designing person-centered referral pathways; the development of digital platforms must prioritize accessibility to reduce digital exclusion among vulnerable groups. At the policy level, integrating DSP into the healthcare system requires a complementary regulatory framework, sustainable financing models, and strict data governance. Developing standardized implementation guidelines is crucial for ensuring that DSP can be widely applied while safeguarding data privacy and fairness.
