# Supplementary figures and images for: Digital social prescribing: a concept analysis
Source: Front Public Health. 2026 Jul 1;14:1857845. doi: 10.3389/fpubh.2026.1857845 (PMC13369115; doi:10.3389/fpubh.2026.1857845)

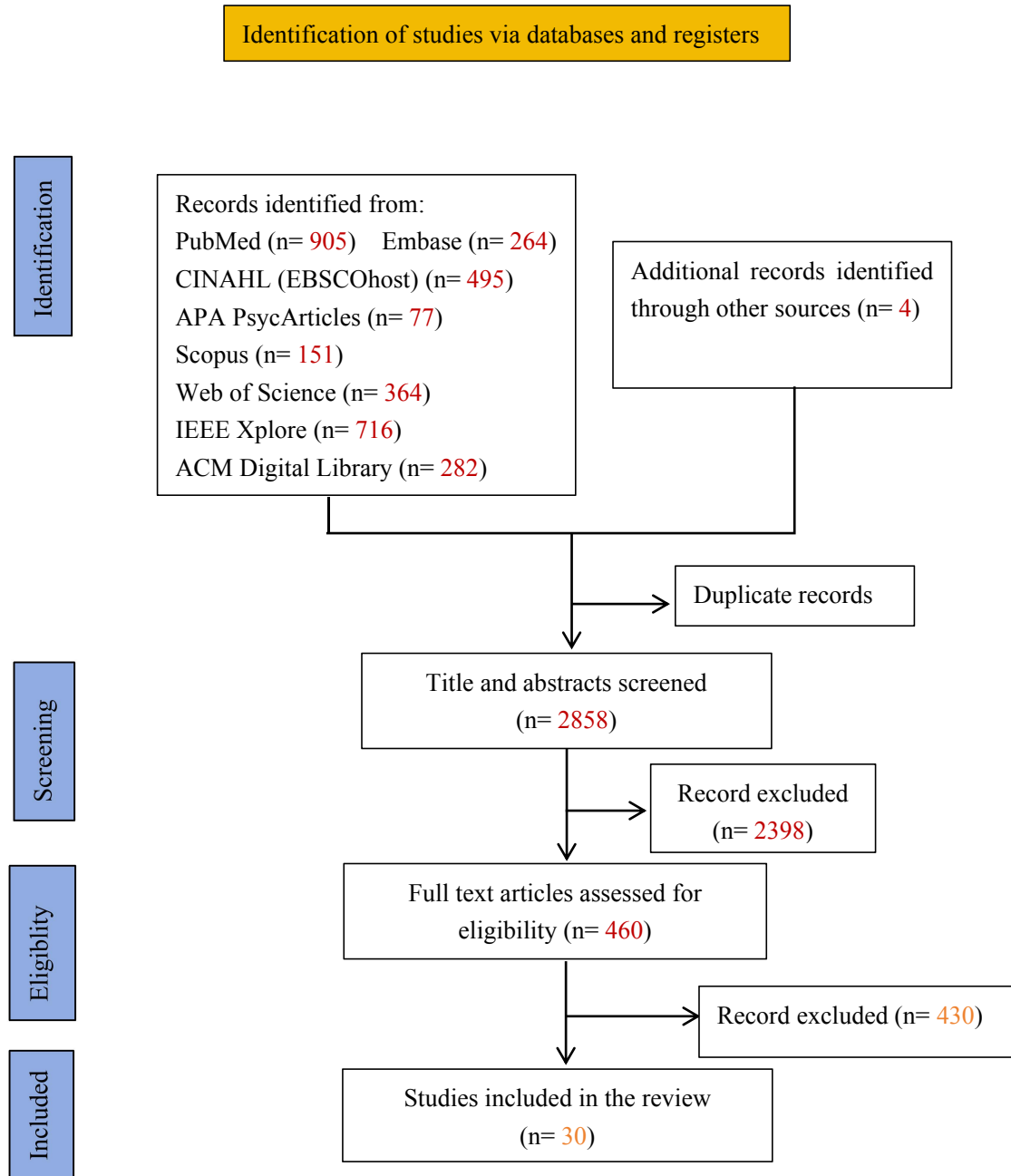

Supplementary Figure1. PRISMA flow diagram for the selection process.

Supplement: Supplementary file 1 [file Data_Sheet_1.PDF]

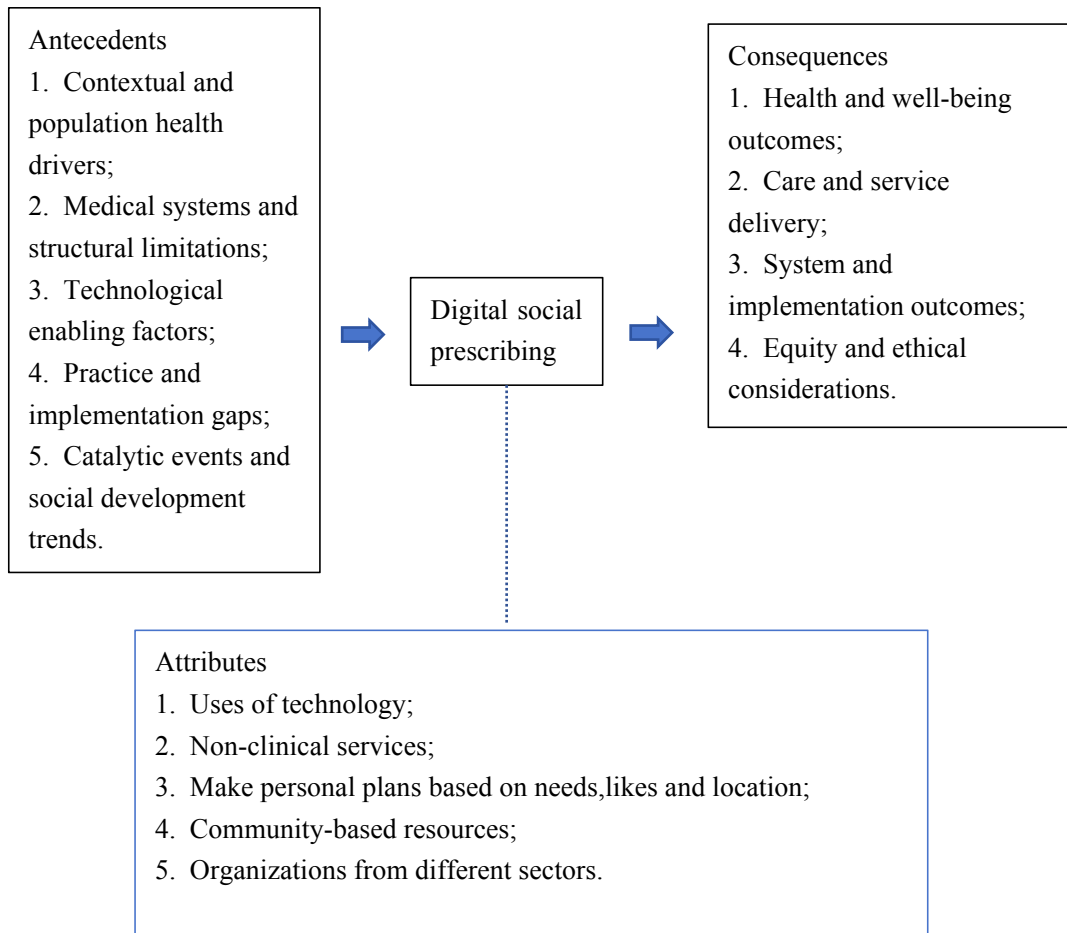

Supplementary Figure 2 Concept analysis of Digital social prescribing

Supplement: Supplementary file 2 [file Data_Sheet_2.PDF]
