# Supplementary material for: Digital social prescribing: a concept analysis
Source: Front Public Health. 2026 Jul 1;14:1857845. doi: 10.3389/fpubh.2026.1857845 (PMC13369115; doi:10.3389/fpubh.2026.1857845)
Supplement: Supplementary file 3 [file Data_Sheet_3.PDF]

Table 1 Search strategy

	Pubmed:	limit
#1	"Telemedicine"[Mesh] OR "Digital Technology"[Mesh] OR "Medical Informatics"[Mesh] OR "Electronic Health Records"[Mesh] OR (electronic health[tiab]) OR (digital medicine*[tiab]) OR (digital health*[tiab]) OR (eHealth*[tiab]) OR (Web-based[tiab]) OR (mHealth*[tiab]) OR (telehealth*[tiab]) OR (telemedicine*[tiab]) OR (internet-based[tiab]) OR (mobile app*[tiab]) OR (e-health*[tiab]) OR (m-health*[tiab])	Filters: Written in the English Language, Humans, Exclude preprints, Studies were published between The database will be established to May 2026, Full-text publications.
#2	"Social Prescribing"[Mesh] OR (social prescribing[tiab]) OR (social prescription*[tiab]) OR (social prescribe[tiab]) OR (social prescribed[tiab]) OR (community referral*[tiab]) OR (non-medical referral*[tiab]) OR (nonmedical referral*[tiab]) OR (social referral*[tiab]) OR (social care referral*[tiab]) OR (link worker*[tiab]) OR (community navigat*[tiab]) OR (community linkag*[tiab]) OR (GP referral*[tiab])	
#3	#1 AND #2	
	CINAHL (EBSCOhost)	limit
#1	(MH "Digital Technology"+) OR (MH "Computers"+) OR (MH "Technology"+) OR (TI "digital health*" OR AB "digital health*") OR (TI "electronic health*" OR AB "electronic health*") OR (TI "ehealth*" OR AB "ehealth*") OR (TI "mhealth*" OR AB "mhealth*") OR (TI "digital medicine" OR AB "digital medicine") OR (TI "digital media" OR AB "digital media") OR (TI "virtual microscop*" OR AB "virtual microscop*") OR (TI "telehealth*" OR AB "telehealth*") OR (TI "telemedicine*" OR AB "telemedicine*")	Filters: Written in the English Language, Humans, Exclude preprints, Studies were published between The database will be established to May 2026, Full-text publications.
#2	(MH "Social Prescribing") OR (MH "Community Health Services") OR (TI "social prescribing" OR AB "social prescribing") OR (TI "social prescription*" OR AB "social prescription*") OR (TI "social prescribe*" OR AB "social prescribe*") OR (TI "community referral*" OR AB "community referral*") OR (TI "non-medical referral*" OR AB "non-medical referral*") OR (TI "nonmedical referral*" OR AB "nonmedical referral*") OR (TI "social referral*" OR AB "social referral*") OR (TI "social care referral*" OR AB "social care referral*") OR (TI "link worker*" OR AB "link worker*") OR (TI "community navigat*"	

	OR AB "community navigat*") OR (TI "community linkag*" OR AB "community linkag*") OR (TI "general practice referral*" OR AB "general practice referral*") OR (TI "primary care referral*" OR AB "primary care referral*")	
#4	#1 AND #2	
	APA PsycArticles	limit
#1	TI ("digital health*" OR "electronic health*" OR "digital medicine" OR ehealth* OR "e-health*" OR mhealth* OR "m-health*" OR telehealth* OR "tele-health*" OR telecare* OR telemedicine* OR "web-based*" OR "internet-based*" OR "mobile app*") OR AB ("digital health*" OR "electronic health*" OR "digital medicine" OR ehealth* OR "e-health*" OR mhealth* OR "m-health*" OR telehealth* OR "tele-health*" OR telecare* OR telemedicine* OR "web-based*" OR "internet-based*" OR "mobile app*")	Filters: Written in the English Language, Humans, Exclude preprints, Studies were published between The database will be established to May 2026, Full-text publications.
#2	TI ("social prescribing" OR "social prescription*" OR "social prescribe*" OR "socially prescribed" OR "community referral*" OR "non-medical referral*" OR "nonmedical referral*" OR "social referral*" OR "social care referral*" OR "link worker*" OR "community navigat*" OR "community linkage*" OR "care navigation" OR "e-referral*") OR AB ("social prescribing" OR "social prescription*" OR "social prescribe*" OR "socially prescribed" OR "community referral*" OR "non-medical referral*" OR "nonmedical referral*" OR "social referral*" OR "social care referral*" OR "link worker*" OR "community navigat*" OR "community linkage*" OR "care navigation" OR "e-referral*")	
#3	#1 AND #2	
	Embase	limit
#1	'telemedicine'/exp OR 'telehealth'/exp OR 'digital health'/exp OR 'electronic health':ti,ab,kw OR 'e-health':ti,ab,kw OR 'ehealth*':ti,ab,kw OR 'tele-health*':ti,ab,kw OR 'telehealth*':ti,ab,kw OR 'digital health*':ti,ab,kw OR 'digital medicine*':ti,ab,kw OR 'digital intervention*':ti,ab,kw OR 'web-based':ti,ab,kw OR 'internet-based':ti,ab,kw OR 'mhealth*':ti,ab,kw OR 'm-health*':ti,ab,kw OR 'mobile app*':ti,ab,kw	Filters: Written in the English Language, Humans, Exclude preprints, Studies were published between The database will be established to May 2026, Full-text publications.
#2	'social prescribing'/exp OR 'social prescribing':ti,ab,kw	

	OR 'social prescription*':ti,ab,kw OR 'social prescribe*':ti,ab,kw OR 'socially prescribed':ti,ab,kw OR 'social prescribing scheme*':ti,ab,kw OR 'community referral*':ti,ab,kw OR 'non-medical referral*':ti,ab,kw OR 'nonmedical referral*':ti,ab,kw OR 'social referral*':ti,ab,kw OR 'social care referral*':ti,ab,kw OR 'link worker*':ti,ab,kw OR 'community navigat*':ti,ab,kw OR 'community linkage*':ti,ab,kw OR 'e-referral*':ti,ab,kw	
#3	#1 AND #2	
	Scopus	limit
#1	TITLE-ABS-KEY (("electronic health" OR "electronic health" OR "e-health*" OR "ehealth*" OR "m-health*" OR "mhealth*" OR "digital health*" OR "digital medicine*" OR "tele-health*" OR "telehealth*" OR "tele-care*" OR "telecare*" OR "telemedicine*" OR "web-based*" OR "internet-based*" OR "mobile app*"))	Filters: Written in the English Language, Humans, Exclude preprints, Studies were published between The database will be established to May 2026, Full-text publications.
#2	TITLE-ABS-KEY (("social prescribing" OR "social prescription*" OR "social prescribe*" OR "community referral*" OR "social referral*" OR "link worker*" OR "community navigat*" OR "community linkage*" OR "care navigation" OR "e-referral*"))	
#3	#1 AND #2	
	Web of Science	limit
#1	TS=("e-health*" OR "ehealth*" OR "electronic health" OR "m-health*" OR "mhealth*" OR "digital health*" OR "digital medicine*" OR "tele-health*" OR "telehealth*" OR "tele-care*" OR "telecare*" OR "telemedicine*" OR "tele-intensive*" OR "tele-referral*" OR "virtual medicine*" OR "online*" OR "web-based*" OR "internet-based*" OR "mobile app*" OR "technolog*")	Filters: Written in the English Language, Humans, Exclude preprints, Studies were published between The database will be established to Mayl 2026, Full-text publications.
#2	TS=("social prescribing" OR "social prescription*" OR "social prescribe*" OR "socially prescribed" OR "community referral*" OR "non-medical referral*" OR "nonmedical referral*" OR "social referral*" OR "social care referral*" OR "link worker*" OR "community navigat*" OR "community linkag*" OR "general practice referral*" OR "primary care referral*" OR "GP referral*")	
#3	#1 AND #2	
	IEEE Xplore	limit
#1	("All Metadata":ehealth* OR "All	Filters: Written in the English

	Metadata":"e-health*" OR "All Metadata": "digital medicine" OR "All Metadata": "electronic health" OR "All Metadata":mhealth* OR "All Metadata":"m-health*" OR "All Metadata":telehealth* OR "All Metadata":"tele-health*" OR "All Metadata":telecare* OR "All Metadata":"tele-care*" OR "All Metadata":telemedicine* OR "All Metadata":"web-based*" OR "All Metadata":"digital health" OR "All Metadata":"digital healthcare" OR "All Metadata":"digital intervention" OR "All Metadata":"digital interventions" OR "All Metadata":"mobile app" OR "All Metadata":"mobile apps")	Language, Humans, Exclude preprints, Studies were published between The database will be established to May 2026, Full-text publications.
#2	("All Metadata":"social prescribing" OR "All Metadata":"social prescription" OR "All Metadata":"social prescriptions" OR "All Metadata":"social prescribing scheme" OR "All Metadata":"community referral" OR "All Metadata":"community referrals" OR "All Metadata":"social referral" OR "All Metadata":"social referrals" OR "All Metadata":"link worker" OR "All Metadata":"link workers" OR "All Metadata":"community navigator" OR "All Metadata":"community navigators" OR "All Metadata":"care coordination" OR "All Metadata":"care navigation" OR "All Metadata":"navigation system" OR "All Metadata":"navigation systems" OR "All Metadata":"e-referral" OR "All Metadata":"e-referrals")	
#3	#1 AND # 2	
	ACM Digital Library	limit
#1	Abstract:(digital medicine OR internet-based OR ehealth OR electronic health OR telemedicine OR "digital health" OR online OR telehealth OR web-based OR mhealth OR virtual)	Filters: Written in the English Language, Humans, Exclude preprints, Studies were published between The database will be established to May 2026, Full-text publications.
#2	Abstract:("social prescribing" OR "social prescription*" OR "social prescribe*" OR "socially prescribed" OR link worker* OR referral* OR non-medical referral* OR social referral*)	
#3	#1 AND #2	
