# Supplementary material for: Digital social prescribing: a concept analysis
Source: Front Public Health. 2026 Jul 1;14:1857845. doi: 10.3389/fpubh.2026.1857845 (PMC13369115; doi:10.3389/fpubh.2026.1857845)
Supplement: Supplementary file 4 [file Data_Sheet_4.PDF]

Supplementary Table 2 Summary of articles on “Digital Social Prescribing”

	Title	Author Publication Year	Country	Journal Quartile	Citation Count	JIF
Patel et al. (2021)	Opportunities and Challenges for Digital Social Prescribing in Mental Health: Questionnaire Study	2021	UK and Canada	Q1	26	1.59
Health Innovation Network (2019)	How to introduce a social prescribing champion model and/or a digital platform to support your social prescribing offer	2019	UK	Not Applicable		Not Applicable
Harrington et al. (2020)	Digitally enabled social prescriptions: adaptive interventions to promote health in children and young people	2020	UK	Q1	10	2.45
Galway et al. (2019)	Adapting Digital Social Prescribing for SuicideBereavement Support: The Findings of a Consultation Exercise to Explore the Acceptability of Implementing Digital Social Prescribing within an Existing Postvention Service	2019	UK	Q2	15	Not Applicable
Jungmann et al. (2020)	Using technology-enabled social prescriptions to disrupt healthcare	2020	South Africa, Germany, UK	Q1	4	2.45
Moya-Gale et al. (2025)	Stronger Together: A Qualitative Exploration of Social Connectedness in Parkinson’s Disease in the Digital Era	2024	US	Q1	10	1.3
Lee et al. (2022)	Evaluating the Effectiveness of Rural Digital Social Prescribing in Korea:	2023	Korea	Q3	5	Not Applicable

	Protocol for a Cohort Study					
Sandhu et al. (2022)	Developing a research agenda for social prescribing in the UK using lessons from the US	2022	UK	Q1	9	2.36
Rafiei et al. (2025)	The potentials of digital technology in social prescribing: a qualitative study of key stakeholders' perspectives	2025	UK	Q1	3	1.18
Wallace et al. (2020)	Understanding social prescribing in wales: A mixed methods study	2021	UK	Not Applicable	11	
Menhas et al. (2026)	Thematic analysis of how general practitioners perceive digital social prescribing as an intervention aiming at promoting psychosocial health and wellbeing in older adults	2026	UK and Pakistan	Q1	0	1.06
Haynes et al. (2025)	Examining the social prescription process: Barriers, facilitators, and the role of health information technology	2025	US	Q1	0	1.64
Nah et al. (2024)	Advancing social prescribing in Singapore: an update on progress	2024	Singapore	Q1	3	2.48
Wang & Yu (2023)	Strengthening implementation research on social prescribing in mental healthcare for older adults in Western Pacific Region	2023	China	Q1	17	2.48
Gottlieb et al. (2018)	Advancing Social Prescribing with Implementation	2018	US	Q2	66	1.48

	Science					
Menhas et al. (2023)	Community-based social healthcare practices in China for healthy aging:a social prescription perspective analysis	2023	China	Q1	26	1.06
Pola-Garcia et al. (2024)	Sex differences in formal recommendation of assets for health (social prescribing) in Aragon	2024	Spain	Q1	0	1.18
Fu et al. (2024)	Impact of pandemics on primary care: changes in general practitioner antidepressant prescriptions and mental health referrals during lockdowns in England, UK	2024	UK	Q2	3	1.01
Nwadiugwu (2021)	Multi-Morbidity in the Older Person:An Examination of Polypharmacy and Socioeconomic Status	2021	UK and US	Q1	35	Not Applicable
McCulloh et al. (2024)	Reach, Adoption, Implementation, and Sustainability of the Mobile Health for Migrant Health (mHealth-4-Mhealth) Program: Nebraska, 2022–2023	2024	US	Q1	1	1.98
Rogers et al. (2022)	A Local Perspective into Electronic Health Record Design, Integration, and Implementation of Screening and	2022	US	Q3	46	Not Applicable

	Referral for Social Determinants of Health					
Jani et al. (2020)	Investing resources to address social factors affecting health: the essential role of social prescribing	2020	UK	Q1	2	2.45
Corbie-Smith et al. (2019)	Connecting Dots to Bridge the Health Disparities Gap: Implementation of a Scalable Electronic Medical Record–Integrated Community Referral Intervention at the Clinic Visit	2019	US	Q1	1	1.98
Bolen et al. (2025)	The Impact of a Bidirectional Clinic to Community Social Care Referral Program	2025	US	Q1	1	1
Gibson et al. (2026)	Expanding Implementation of a Social Determinants of Health Screening and Referral Program to Community–Based Pediatric Clinics	2026	US	Q1	20	1.43
Haynes et al. (2025)	A Human-Centered Approach for Designing a Social Care Referral Platform	2025	US	Q2	1	0.54
Bone et al. (2026)	Using electronic health records to evaluate a children and young people's social prescribing service: challenges and implications for research and practice	2026	UK	Q1	30	1.43
Zhao et al.	Integrating Community and Digital	2026	Korea	Q1	83	1.12

(2026)	Support Through Social Prescribing to Improve Mental Health in Rural Older Adults in South Korea: Quasi-Experimental Study					
Lee et al. (2023)	Global Trends in Social Prescribing: Web-Based Crawling Approach	2023	Worldwide	Q1	14	1.59
Tong et al. (2024)	Comparing cognitive behavioral therapy and social prescribing in patients with loneliness on long-term opioid therapy to reduce opioid misuse: protocol for a randomized controlled trial	2024	US	Q1	54	1.07
