# Supplementary material for: Digital social prescribing: a concept analysis
Source: Front Public Health. 2026 Jul 1;14:1857845. doi: 10.3389/fpubh.2026.1857845 (PMC13369115; doi:10.3389/fpubh.2026.1857845)
Supplement: Supplementary file 5 [file Data_Sheet_5.PDF]

Supplementary Table 3 Attributes

Attributes	Original description	Reference
Uses of technology	**Technologies** are currently being used to provide support , primarily through the use of management platforms, as well as apps	Harrington et al. (2020)
	Any **technology**, information or electronic system that enables social prescribing, the use of this **technology** for social prescription could offer a health benefit to our modern society	Patel et al. (2021)
	**Using digital technology** to support social prescribing	Health Innovation Network (2019)
	**Software platforms** provide the intelligence to manage all of the services, users	Galway et al. (2019)
	**Technology platform** that produces a personalised list of community resources from data in the patient	Lee et al. (2022)
	In **technology** platforms that facilitate referrals from the health sector	Sandhu et al. (2022)
	Involves the use of any technology that facilitates social prescribing	Moya-Gale et al. (2025)
	Information **technology** is seen as a tool for facilitating the social prescription process	Haynes et al. (2025)
	Social prescribing service providers use the **platform**	Lee et al. (2023)
	Are enabled by connected **intelligence**, connected devices, user interfaces, the cloud and network connectivity combined with data	Jungmann et al. (2020)
	**Using a digital directory and digital platform** across pathways will ensure that social prescribing	Wallace et al. (2020)
	**A digital version** of social prescribing, is a promising development that may benefit the geriatric population	Menhas et al. (2026)
	**Electronic** Social Prescribing, elderly patients use some of the basic features of **smartphones and some applications** to ease their return to the community upon discharge	Nah et al. (2024)
	**Digital platform** is expected to produce a personalized psychosocial interventions list from data of **computerized** assessment and	Wang & Yu (2023)

	share the list among social prescribing implementation team	
	Use **electronic health records** (EHRs) to support point-of-care applications	Gottlieb et al (2018)
	**E-Social prescription** aims to enhance individuals' overall health and well-being by fostering social support	Menhas et al. (2023)
	For its **application, a Social Prescription Protocol** is included in the Electronic Health Record and a searcher of health assets helps to visualise the existing assets and choose the most adequate	Pola-Garcia et al. (2024)
	Considerable number of SP services were able to rapidly transition to **digital platforms**	Fu et al. (2024)
	The use information from **electronic health records** and social prescribing to prevent unnecessary drug prescription	Nwadiugwu (2021)
	Families use the **mHealth tool** for daily symptom screening to assess household risk	McCulloh et al. (2024)
	We custom designed and built into the **Epic EHR a SDOH screening tool** integrated with a community resource network management (CRNM) software-as-a-service (SaaS) platform	Rogers et al. (2022)
	compare the effectiveness of two group-based, **telehealth-delivered interventions** with treatment as usual	Tong et al. (2024)
	an **electronic medical record–integrated intervention** that uses algorithms to systematically and automatically match people	Corbie-Smith et al. (2019)
	The referral order in the **EHR** triggered the sending of a continuity of care document (CCD) via Direct messaging through the **Health Information Exchange (HIE)** to UW 211	Bolen et al. (2025)
	The SDOH program, which is built into the **electronic health record (EHR)**	Gibson et al. (2026)
	The **algorithm implemented** in the system... ranks and matches available programs to patients based on program eligibility criteria	Haynes et al. (2025)
	Records were extracted from **Joy, an online platform** for managing SP	Bone et al. (2026)
	social prescribing services are provided... through the **digital platform**	Lee et al. (2023)
	The education plan included basic **smartphone operation** methods to use apps most commonly	Zhao et al. (2026)

	used	
Non-clinical services	Through referrals to **non-clinical services** such as exercise and dance classes, arts communities, peer support networks, and legal and financial services	Harrington et al. (2020
	Use electronic patient records and community directory software to match **nonmedical activities**	Patel et al. (2021)
	Is a model of support that connects people to a range of **non-medical** community-based services via a link worker	Galway et al. (2019)
	Connect patients with community-based services to address **non-medical** needs	Sandhu et al. (2022)
	Through link workers who have been trained in using community resources **rather than the clinical medical** system	Lee et al. (2023)
	Address **non-biomedical** issues related to social determinants of health	Jungmann et al. (2020)
	Connects individuals with **nonmedical** support within their communities	Rafiei et al. (2025)
	Through which older adults **can access nonclinical services** and support	Menhas et al. (2026)
	A mechanism for linking patients with **non-medical sources** of support within the community	Fu et al (2024)
	connect people with **non-medical forms** of support	Bone et al. (2026)
	prescribe **nonpharmacological** interventions to tackle social loneliness	Lee et al. (2023)
	linking individuals to **nonmedical** community resources.	Zhao et al. (2026)
	through activities such as **sports and leisure/arts** and can address social factors through activities	Jani et al. (2020)
Make personal plans based on needs, likes and location	Takes into account the **needs** of the **individual** and knowledge of **local services**	Harrington et al. (2020)
	The matching process involves using an algorithm designed to match activities to a patient based on their **preferences**, **needs**, and **locality**	Patel et al. (2021)

	In response to these **individual** social health needs, healthcare systems may screen **individuals** for social needs	Haynes et al. (2025)
	Be **personalised** and be highly adaptable to **individual needs**/expectations	Jungmann et al. (2020)
	Allows professionals to refer **individuals** to community based services that address social, emotional, and practical **needs** impacting health, to focus on those with the greatest need	Rafiei et al. (2025)
	Delivered **person-centered** mental healthcare, including assessing mental health needs and resources of social support, developing and implementing **individualized mental healthcare plan, and evaluating the feedback** of older adults	Wang & Yu (2023)
	To address **identified needs**	Gottlieb et al. (2018)
	support within their community, based on their **values and preferences... to develop a personalised care plan**	Bone et al. (2026)
	to co-create a **person-centered** care plan	Rogers et al. (2022)
	curated list of programs that **fit them the best**	Haynes et al. (2025)
	comprehensive model of **personalized** care	Lee et al. (2023)
	Integrating **person-centered** principles into	Zhao et al. (2026)
	community resources the **patient might need**	Bolen et al. (2025)
Community-based resources	Working across Voluntary **Community**	Harrington et al. (2020)
	Are typically offered by voluntary and **community** sector organizations	Patel et al. (2021)
	Social welfare by connecting them to **community services**	Health Innovation Network
	Is a model of support that connects people to a range of non-medical **community-based services** via a link worker	Galway et al. (2019)
	Produces a personalised list of **community resources** from data in the patient	Sandhu et al. (2022)
	Provide connections or referrals to **community-based** social service organizations	Haynes et al. (2025)

	Through link workers who have been trained in using **community resources** rather than the clinical medical system	Lee et al. (2023)
	To connect individuals to **sources** of support within their **community**	Jungmann et al. (2020)
	Linking health professionals with **community services**	Rafiei et al. (2025)
	This enhances **community resource** awareness and **access**	Nah et al. (2024)
	**Online healthcare communities** to promote social connect	Menhas et al (2023)
	A mechanism for linking patients with non-medical **sources** of support within the **community**	Fu et al. (2024)
	to assist with identifying and accessing **community resources**	McCulloh et al. (2024)
	with identified social needs to **social service resources in their community**	Rogers et al. (2022)
	linking patients in primary care with **sources** of support within the **community** to help improve their health and well-being	Jani et al. (2020)
	connecting people to the **resources available in their community**	Corbie-Smith et al. (2019)
	UW 211 referred patients to assess and connect them with **community resources**	Bolen et al. (2025)
	a list of **community resources** near the patient's home zip code	Gibson et al. (2026)
	help providers connect patients with **community resources** to meet health-related social needs	Haynes et al. (2025)
	a strategy to connect **community resources** to address social loneliness at the state level	Lee et al. (2023)
	to refer patients to various **community-based** activities and services	Zhao et al. (2026)
Organizations from different sectors	Provided by **charities, volunteer communities** and other **thirdsector organisations**.	Harrington et al. (2020)
	**Initiatives, health, social care and voluntary organisations** can join up all their sources of support	Health Innovation Network
	Their details are passed to public health bodies, including the **NHS** and **voluntary** and **community** sector providers, commissioned to	Galway et al. (2019)

	provide counselling	
	Are recommended by **multiple** US professional organisations, and have been incentivised by policymakers and payers	Sandhu et al. (2022)
	Social prescribing has emerged as an approach that enables the link social care, mental health professionals, **social workers, community staff, and volunteers formulated a coordinated service team**	Wang & Yu (2023)
	Such as **charities, the voluntary sector, and community groups**. It provides patients with additional sources of support	Fu et al (2024)
	Addressing SDoH requires **focused and coordinated action across multiple sectors**	Haynes et al. (2025)
	based on the understanding of roles and the impact of **multisectoral partnerships**	Lee et al. (2023)
	various community-based activities and services, such as... which **voluntary and community organizations provide**.	Zhao et al. (2026)
