# Supplementary material for: Digital social prescribing: a concept analysis
Source: Front Public Health. 2026 Jul 1;14:1857845. doi: 10.3389/fpubh.2026.1857845 (PMC13369115; doi:10.3389/fpubh.2026.1857845)
Supplement: Supplementary file 6 [file Data_Sheet_6.PDF]

Supplementary Table 4 Antecedents

Antecedent Category	Original description	Reference
Contextual and population health drivers	Health inequality and poverty driven by social, environmental and economic differences also plays a significant role in these age groups and can have a large impact	Harrington et al. (2020)
	consult on the acceptability and potential value of DSP for addressing the complexities of suicide bereavement support.	Galway et al. (2019)
	This article is in our series on social prescriptions Healthcare systems are facing increasing need and demand because of aging populations, increasing chronic disease and multimorbidity	Jungmann et al. (2020)
	The intertwining of the pervasive reality of social isolation in older adults and the effects of the COVID-19 pandemic has been referred to as a synergy of pandemics	Moya-Gale et al. (2025)
	The aging of the global population presents the need to introduce various medical systems.	Lee et al. (2022)
	staff refer patients to 'link workers' who connect patients with community-based services to address non-medical needs	Sandhu et al. (2022)
	Worldwide, health and social care systems face increasing demand due to population ageing, rising chronic disease prevalence, and the emergence of advanced diagnostic and treatment technologies.	Rafiei et al. (2025)
	ocial prescribing in Wales is defined as 'connecting citizens to community support to better manage their health and well-being	Wallace et al. (2020)
	Population aging has intensified the need for interventions that address the psychosocial health and wellbeing of older adults. Digital social pre-scribing	Menhas et al. (2026)
	his patient population comprises only 5 % of the total patient population but accounts for approximately 50 % of healthcare costs	Haynes et al. (2025)
	Social prescribing, emerged as an innovative approach linking medical and social care, has been observed to potentially benefit mental wellbeing of older adults	Wang & Yu (2023)
	Social prescribing (SP) is an important tool... to help to reduce health inequities.	Gottlieb et al. (2018)
	The global population is aging, and the number of	Menhas et al.

	people suffering from chronic diseases is increasing.	(2023)
	With 22.5% of the population over 65, Aragon has an ageing population.	Pola-Garcia et al. (2024)
	The COVID-19 pandemic and subsequent nationwide lockdowns have had a significant impact on mental health	Fu et al. (2024)
	people with multi-morbidity usually face social, mental, and physical health conditions that require care from multiple services, and are often in need of support	Nwadiugwu (2021)
	Migrant families face challenges to health and well-being from COVID-19	McCulloh et al. (2024)
	Loneliness... has increased substantially during the COVID-19 pandemic to greater than half of U.S. adults	Tong et al. (2024)
	recognize the risks of unmet health-related social needs	Corbie-Smith et al. (2019)
	a sharp rise in the aging population, noncontagious diseases, and mental health problems	Lee et al. (2023)
Medical systems and structural limitations	health and care services across the UK are facing huge challenges to meet the weight of demand, often with less resources than ever before	Health Innovation Network (2019)
	one of the main challenges is the connecting of information between different organisations and connecting different systems to enable those involved in SP to best serve people and communities	Galway et al. (2019)
	New pharmaceuticals must significantly outperform the gold standard to justify the high prices charged to recoup their development costs, which typically lie in the billions	Jungmann et al. (2020)
	Many services— including the National Health Service (NHS)—struggle with insufficient sustainable funding to meet the popula-tion's complex needs	Rafiei et al. (2025)
	Concerns were expressed throughout the study about the 'fundamental problem of the funding model' and the challenges for sustainability that this brings	Wallace et al. (2020)
	Under the current biomedical orientation in primary care, physical health problems are prioritized over psychosocial problems	Menhas et al. (2026)
	Few healthcare systems have tools or workflows	Haynes et al.

	that systematically collect individual needs and provide reliable referrals to Community-Based Organizations (CBOs)	(2025)
	The response to mental healthcare needs is often fragmented and poorly coordinated, especially in Western Pacific Region	Wang & Yu (2023)
	although non-clinical community-based social healthcare practices in China effectively promote healthy aging, there is a shortage of information on their applicability and effectiveness, especially from a social prescription perspective	Menhas et al. (2023)
	The pandemic has also disrupted the usual practices of mental health services, there were substantial disruptions to the provision of face-to-face mental health services.	Fu et al. (2024)
	continuous spending on patients with multi-morbidity is unsustainable and there is a need for a more holistic approach to the complexities of multi-morbidity	Nwadiugwu (2021)
	over-reliance on the biomedical model	Jani et al. (2020)
	Limited time, professional training	Bolen et al. (2025)
	barrier for health care systems is the complex and often non-standardized social care referral process	Haynes et al. (2025)
	have yet to use the full potential of informatics, data science, and technology	Corbie-Smith et al. (2019)
	the clinical approach alone cannot solve these problems	Zhao et al. (2026)
	counties are designated health care shortage areas	McCulloh et al. (2024)
	ue to limited resources and implementation barriers	Gibson et al. (2026)
	lack of appropriate sensitive and standard measures, little guidance... fragmented systems and time constraints	Bone et al. (2026)
Technological enabling factors	Digital technologies have become increasingly pervasive within the society	Patel et al. (2021)
	Digital technologies are also currently being used to support social prescriptions, primarily through the use of management platforms, as well as apps and digital therapeutics.	Harrington et al. (2020)
	Korea is a digital powerhouse... with the highest	Lee et al.

	internet penetration rate of 99.9% as of 2021	(2022)
	Technological advances and new devices also enhance service quality by analysing diverse patient information... to tailor personalised care plans	Rafiei et al. (2025)
	China has over 1 billion Internet users, with a digital divide among older adults	Menhas et al. (2026)
	These efforts may facilitate utilization of collected data in the living environment and save workforce and time costs for implementing social prescribing schemes	Wang & Yu (2023)
	including the workforce and technologic infrastructure needed for intersectoral work	Gottlieb et al. (2018)
	Automation, artificial intelligence, cloud technology, and the new generation of digital technologies are advancing quickly.	Menhas et al. (2023)
	ntegration within the electronic health record system provides opportunities for health care staff to support their patients more easily	Haynes et al. (2025)
	Mobile health (mHealth) technologies can overcome geographic barriers to accessing high-quality health care information	Rogers et al. (2022)
	Most primary care clinics have the information technology infrastructure for these referrals,	Bolen et al. (2025)
Practice and implementation gaps	However, Social Prescribing and link workers are not available in all GP surgeries and are often over-burdened when they are	Health Innovation Network (2019)
	Lack of evidence Lack of data on the effectiveness of digital SP One of the challenges that has been mentioned by a few study participants was lack of evidence around the impact and effectiveness of digital social prescribing	Rafiei et al. (2025)
	While technologies have been developed, a major barrier is that the social prescription process is poorly understood.	Haynes et al. (2025)
	it remained uncertain how to successfully implement social prescribing schemes in realistic world, such as community	Wang & Yu (2023)
	Although non-clinical community-based social healthcare practices in China effectively promote healthy aging, there is a shortage of information on their applicability and effectiveness	Menhas et al. (2023)
	The higher rate of SP referrals for ethnic minorities gives rise to concerns regarding equity in mental	Fu et al. (2024)

	health service provision.	
	The use of the EHR system... is still lacking, and examples for customizing the EHR to meet the workflows... are missing.	Rogers et al. (2022)
	at the expense of interventions that can address social factors related to health	Jani et al. (2020)
	the primary function of most EMRs is to facilitate documentation and billing... We have yet to use the full potential of informatics	Corbie-Smith et al. (2019)
	we lack full data on whether resources were used by patients	Bolen et al. (2025)
	gaps remain in understanding of the best implementation strategies and infrastructure	Gibson et al. (2026)
	esults in a gap in actionable information	Haynes et al. (2025)
	it difficult to be managed by a single team	Lee et al. (2023)
	it was challenging to fully implement all elements recommended by established intervention guidelines	Zhao et al. (2026)
Catalytic events and social development trends	The way we live our lives is changing, including the way we access information and services.	Health Innovation Network (2019)
	Payment reform and quality measures' influence on social prescribing The shift from fee-for-service to value-based payment models in the US has created financial incentives to adopt interventions	Sandhu et al. (2022)
	The pandemic has worsened this problem, with mandatory distancing causing a 28.1% increase in psychological distress worldwide.	Menhas et al. (2026)
	With the recent launch of 'Healthier SG' program, a nation-wide initiative which promotes preventive care and has a SP component	Nah et al. (2024)
	Growing awareness of the role of social factors in shaping health... has fueled a wave of experimentation.	Gottlieb et al. (2018)
	In recent times, the world has witnessed unprecedented challenges, with the COVID-19 pandemic being a major disruptor to various aspects of daily life	Fu et al. (2024)
	Recent healthcare delivery system reforms have spurred health policy innovations that have placed	Rogers et al. (2022)

	increasing emphasis on healthcare systems to respond to SDOH	
	ighlighted by rising inequalities and decreasing life expectancies in high-income countries including the UK	Jani et al. (2020)
	Due to the increased recognition of the contribution of SDoH to health outcomes and health equity, primary care clinics have begun exploring ways to screen	Bolen et al. (2025)
	a global national policy, as the world is witnessing a sharp rise in the aging population, noncontagious diseases, and mental health problems	Lee et al. (2023)
	now experiencing super-aging speed	Zhao et al. (2026)
	Services and the National Institutes of Health have recognized the critical role social determinants play	Haynes et al. (2025)
