# Supplementary material for: Digital social prescribing: a concept analysis
Source: Front Public Health. 2026 Jul 1;14:1857845. doi: 10.3389/fpubh.2026.1857845 (PMC13369115; doi:10.3389/fpubh.2026.1857845)
Supplement: Supplementary file 7 [file Data_Sheet_7.PDF]

Supplementary Table 5 Consequence

Consequence	Original description	Reference
Health and well-being outcomes	Improving long-term health outcomes and reducing health inequalities, addressing the environmental and lifestyle-related issues, and technology and data can help to enhance both of these	Harrington et al. (2020)
	To affect our health at much lower cost and fewer side effects than most medicines	Jungmann et al. (2020)
	That support overall well-being and quality of life	Sandhu et al. (2022)
	Improve mental and social well-being	Rafiei et al. (2025)
	Promote participation and reduce social isolation, build social connections and promote psychological health	Menhas et al. (2026)
	The process and allows staff the ability to focus more effort on individuals with greater needs	Haynes et al. (2025)
	Maximize their opportunities for participation, health, and security in to enhance their standard of living, a direct indication of successful aging	Menhas et al. (2023)
	Improves mental health and reduces the burden of mental illness, particularly for people from a disadvantaged background	Fu et al. (2024)
	improve children and young people's (CYP) well-being... improved well-being, reduced loneliness and less healthcare utilisation	Bone et al. (2026)
	effectively reduced depression and increased social support and life satisfaction	Zhao et al. (2026)
	blood pressure control among the referred adult populatio	Bolen et al. (2025)
	improve health outcomes and improve population health	Rogers et al. (2022)
	improving community resilience in public health emergencies	McCulloh et al. (2024)
	promoting self-care and improving management of long-term conditions	Jani et al. (2020)
	fosters mental health, alleviates stress and loneliness	Lee et al. (2023)
	improving the quality of life of patients	Haynes et al. (2025)
Care and service delivery	Can join up all their sources of support, enabling health and social care professionals to have an up to date knowledge of available services, be able to easily refer individuals to activities and monitor	Health Innovation Network (2019)

	the impact on the wider system	
	Expand their options to health care, particularly given the dynamic and ever-changing technological advancements	Moya-Gale et al. (2025)
	Can consult doctors, receive a social prescribing program, communicate with link workers and social prescribing sites, and perform the program	Lee et al. (2022)
	To foster collaboration among healthcare professionals, community leaders, and practitioners dedicated to advance SP for enhancing health and well-being nationwide	Nah et al. (2024)
	a rise in social prescribing referrals, emerging as a valuable resource for psychological support amid heightened mental health strain	Fu et al. (2024)
	Developing patient pathways from a more patient-centered approach rather than using a disease-specific model in multi-morbid disease management for older patients	Nwadiugwu (2021)
	minimize burden on screening staff, and to align with current workflows, thereby making this a standard of care	Rogers et al. (2022)
	Integration into the EHR is considered a crucial component for the program to be delivered efficiently to large numbers of patients in busy clinical settings	Gibson et al. (2026)
	Integration within the electronic health record system provides opportunities for health care staff to support their patients more easily given these barriers	Haynes et al. (2025)
	ink workers to monitor referrals, connect with local services, record appointments and prescriptions, and measure impact	Bone et al. (2026)
	as key connectors between digital and local community resources in sustaining and scaling such programs	Zhao et al. (2026)
	reduce referral barriers and address health-related social needs	Corbie-Smith et al. (2019)
	that could disrupt the paradigm of how health and care are delivered	Jani et al. (2020)
System and implementation outcomes	Result in the process being less costly	Patel et al. (2021)
	Improve patient and population outcomes while optimising resource utilisation	Jungmann et al. (2020)

	Reducing unnecessary medical expenses	Lee et al. (2022)
	Participation had increased since moving to a digital platform.	Wallace et al. (2020)
	Increase the coverage of care service	Wang & Yu (2023)
	easibility and scalability of mHealth-targeted... at-home testing and... surveillance for SDOH challenges	McCulloh et al. (2024)
	positive balance and generating savings for the NHS	Jani et al. (2020)
	An EHR-facilitated, closed-loop CCL program to address patients’ social needs is feasible	Bolen et al. (2025)
	Integration within the electronic health record system provides opportunities for health care staff to support their patients more easily	Haynes et al. (2025)
	innovative, scalable, and resource-efficient intervention	Corbie-Smith et al. (2019)
	This pilot study supports the feasibility... of integrating community and digital support through culturally embedded social prescribing for rural older adults.	Zhao et al. (2026)
Equity and ethical considerations	Offers a potential solution for overcoming the stigma of suicide bereavement by providing connectivity and a safe, knowledge-based support system	Galway et al. (2019)
	To deliver services to those who never had access to them previously	Jungmann et al. (2020)
	Toward aprogressive amalgamation, specifically emphasizing organizations dedicated to primary care networks	Menhas et al. (2023)
	To reduce health inequities. Assets can operate at the individual, group, community, and population level as protective (or promoting) factors to buffer against life’s stresses	Pola-Garcia et al. (2024)
	Whether this trend reflects a genuine preference for or benefit from SP among these populations, or if potentially indicates disparities in access to other forms of mental health support	Fu et al. (2024)
	Reduce social exclusion among older people with multi-morbidity	Nwadiugwu (2021)
	overcome geographic barriers to accessing high-quality health care information and can be adapted for use in multiple languages	McCulloh et al. (2024)

	take actionable steps to achieve health equity and reduce health disparities in healthcare	Rogers et al. (2022)
	Great potential exists to advance health equity by harnessing technology and data in electronic medical records	Corbie-Smith et al. (2019)
	social needs will be critical in advancing health equity	Bolen et al. (2025)
	Addressing SDOH needs allows every person the opportunity to attain their highest level of health	Gibson et al. (2026)
	Addressing SDoH requires focused and coordinated action... to reduce health disparities	Haynes et al. (2025)
	Digital support... was intentionally designed as a low-threshold, supportive component... to enhance accessibility and inclusivity among older rural adults.	Zhao et al. (2026)
	intentionally sample patients of diverse gender, race/ethnicity and geographic location	Tong et al. (2024)
