# Supplementary material for: Digital social prescribing: a concept analysis
Source: Front Public Health. 2026 Jul 1;14:1857845. doi: 10.3389/fpubh.2026.1857845 (PMC13369115; doi:10.3389/fpubh.2026.1857845)
Supplement: Supplementary file 8 [file Data_Sheet_8.PDF]

Supplementary Table 6 Empirical referents

Reference	Empirical referents
Patel et al. (2021)	Interaction logs on dedicated platforms (e.g., Elemental) and digital mood self-monitoring records
Health Innovation Network (2019)	Electronic prescription generation records via HealthUnlocked and response rates to automated follow-up emails.
Harrington et al. (2020)	Usage frequency of integrated clinical plugins and user navigation/clickstream data on mHealth apps (e.g., NHS Go).
Galway et al. (2019)	Electronic assessment data for bereavement support and interaction data from online consensus tools (e.g., Well Sorted)
Jungmann et al. (2020)	24/7 chatbot interaction logs and sensor data from smart wearables (e.g., Apple Watch)
Moya-Gale et al. (2025)	Zoom remote rehabilitation session attendance logs and health interaction data on streaming platforms (e.g., YouTube)
Lee et al. (2022)	Validated scale scores (GDS-K, eHEALS) and statistical outputs from DID economic models
Sandhu et al. (2022)	Waiting-room tablet logs for social needs screening and successful resource connection rates
Rafiei et al. (2025)	Platform-captured health outcome KPIs and coverage density of electronic social care records
Wallace et al. (2020)	Access logs of national digital directories (e.g., DEWIS) and platform e-referral volumes.
Menhas et al. (2026)	Activity of SP functions within general social apps (WeChat) and psychometric scale scores (Loneliness, QoL).
Haynes et al. (2025)	Tablet-based electronic screening records (MSMH) and CBO instant chat logs within the SCH app
Nah et al. (2024)	Dynamic update logs in digital asset portals (LAMP) and validated tool scores (UCLA Loneliness, WHO-5)
Wang & Yu (2023)	Intervention checklist output logs from digital triage platforms and implementation outcome metrics (acceptability, adoption)
Gottlieb et al. (2018)	EHR-integrated PRAPARE social risk assessment records
Menhas et al. (2023)	Interaction frequency in social networking groups (WeChat/QQ) and physiological data uploaded via wearable trackers
Pola-Garcia et al. (2024)	Formal prescription records linked with social diagnostic codes (ICPC Chapter Z) within EHRs
Fu et al. (2024)	EHR-recorded ratios of social to clinical referrals
Nwadiugwu (2021)	Extracted non-medical social data for clinical decision support in EHRs
McCulloh et al. (2024)	Screening frequency, technology adoption and implementation rate, service demand volume
Rogers et al. (2022)	Screening and referral scale

Tong et al. (2024)	Clinical record data, comprehensive health indicators
Jani et al. (2020)	Resource allocation ratio, service coverage breadth
Corbie-Smith et al. (2019)	Information sharing behavior, implementation outcome
Bolen et al. (2025)	Social demand density, referral rate, resolution rate
Gibson et al. (2026)	Plan coverage, implementation strategy adopted
Haynes et al. (2025)	Resource matching accuracy
Bone et al. (2026)	Service utilization intensity, data quality indicators
Lee et al. (2023)	Policy influence, trend data
Zhao et al. (2026)	Satisfaction score
